# The Resurgence of Cyber Racism During the COVID-19 Pandemic and its Aftereffects: Analysis of Sentiments and Emotions in Tweets

**DOI:** 10.2196/19833

**Published:** 2020-10-15

**Authors:** Akash Dutt Dubey

**Affiliations:** 1 Jaipuria Institute of Management Jaipur India

**Keywords:** COVID-19, pandemic, China, racism, WHO, Twitter, infodemiology, infodemic

## Abstract

**Background:**

With increasing numbers of patients with COVID-19 globally, China and the World Health Organization have been blamed by some for the spread of this disease. Consequently, instances of racism and hateful acts have been reported around the world. When US President Donald Trump used the term “Chinese Virus,” this issue gained momentum, and ethnic Asians are now being targeted. The online situation looks similar, with increases in hateful comments and posts.

**Objective:**

The aim of this paper is to analyze the increasing instances of cyber racism during the COVID-19 pandemic, by assessing emotions and sentiments associated with tweets on Twitter.

**Methods:**

In total, 16,000 tweets from April 11-16, 2020, were analyzed to determine their associated sentiments and emotions. Statistical analysis was carried out using R. Twitter API and the sentimentr package were used to collect tweets and then evaluate their sentiments, respectively. This research analyzed the emotions and sentiments associated with terms like “Chinese Virus,” “Wuhan Virus,” and “Chinese Corona Virus.”

**Results:**

The results suggest that the majority of the analyzed tweets were of negative sentiment and carried emotions of fear, sadness, anger, and disgust. There was a high usage of slurs and profane words. In addition, terms like “China Lied People Died,” “Wuhan Health Organization,” “Kung Flu,” “China Must Pay,” and “CCP is Terrorist” were frequently used in these tweets.

**Conclusions:**

This study provides insight into the rise in cyber racism seen on Twitter. Based on the findings, it can be concluded that a substantial number of users are tweeting with mostly negative sentiments toward ethnic Asians, China, and the World Health Organization.

## Introduction

Since their inception, social media networks have served as platforms where people worldwide can express their views and opinions. In 1993, The New Yorker [1] had published a cartoon, titled “On the Internet, nobody knows you're a dog,” signifying that caste, race, ethnicity, religion, and appearance do not matter when you are on the internet. In contrast, Nakamura [2] denied the existence of this utopian model and suggested that the internet is “an outstanding example of a racist medium.” Brown [3] has concluded that the internet has often been a place where racism is disseminated in various ways, including through certain websites. These websites used offensive stereotypes to establish white supremacy over the ethnic peoples of Africa. In 2011, Clark et al [4] analyzed weblogs using modified consensual qualitative research to study different types of racial microaggression targeted at Native Americans. There have been sufficient studies that have verified the presence of racial aggression and hatred toward different races, ethnicities, and religions on the internet.

At present, the world is facing the brunt of COVID-19. COVID-19 infections were first reported in December 2019, when cases of a severe respiratory infection was observed in several patients from Wuhan, Hubei Province. These patients worked in a wholesale fish and seafood market (known as wet markets) [5]. In January 2020, the markets were closed down, and disinfectants were used to sanitize them. On January 7, 2020, researchers isolated a novel coronavirus, now referred to as SARS-CoV-2. Initially, the World Health Organization (WHO) denied the possibility of human-to-human transmission of SARS-CoV-2 on January 11, 2020. However, confirmed cases continued to soar, and on January 30, 2020, the World Health Organization declared COVID-19 a Public Health Emergency of International Concern (PHEIC) and an epidemic. Finally, on March 11, 2020, the WHO declared COVID-19 as a pandemic. Due to the lack of any specific treatments, the WHO recommended self-isolation and lockdown to reduce the spread of COVID-19.

On March 17, 2020, US President Donald Trump posted the following tweet: “The United States will be powerfully supporting those industries, like Airlines and others, that are particularly affected by the Chinese Virus. We will be stronger than ever before!” [6]. The term “Chinese Virus” sparked a series of controversies; hashtags like #ChineseVirus and #WuhanVirus started trending among supporters of Donald Trump [7-9] on various online social networking platforms, with Twitter being the most prominent of them. Racial slurs and profane words against Asian communities have been visible on Twitter ever since [10,11]. In Italy, there have been several reports of anti-Chinese racism and discrimination. It is also believed that the increasing rate of xenophobia in Italy was the result of the circulation of information related to racism [12]. According to Budhwani and Sun [13], there has been a 10-fold increase in the usage of words like “China Virus” and “Chinese Virus.”

This research was conducted keeping in mind that there has been an increase in cyber racism and online displays of hatred during the COVID-19 pandemic. The main aim of this research is to analyze the sentiments and emotions associated with the tweets that mention “Chinese Virus” or “Wuhan Virus.” This research also analyzed the most frequently used words in these tweets.

## Methods

Twitter, one of the world’s most popular microblogging service providers, was launched in 2006. The estimated number of Twitter users is 330 million worldwide. Initially, tweets were limited to 140 characters, but this was later increased to 280 characters. Twitter has been often used as a platform where people disseminate information, as well as share their opinion and emotions. This rapid sharing of opinions enables researchers to determine the sentiments associated with almost everything (eg, sentiments toward products, movies, politics, digital technology, and natural calamities) [14-18].

Sentiment analysis of tweets has also been used to determine the general population’s perspective on different diseases. Sentiment analysis of Twitter posts has been carried out to study the topic coverage and sentiments regarding the Ebola virus [19]. This study separately analyzed two media sources (ie, Twitter and news sources). Similarly, a study was conducted to examine the key topics that influenced negative sentiments on Twitter regarding the Zika virus [20]. Sentiment analysis was also done to analyze tweets by patients who were affected by Crohn disease, to gain an understanding of their perspective on a specific medical therapy [21].

While there is no single accepted psychological theory of basic human emotions, most studies accept the theory that a simple positive-negative dichotomy cannot be used to categorize human emotions as a whole. On the same lines, it is believed that the automatic sentiment analysis must also implement finely tuned algorithms to detail human emotions. Sentimentr (CRAN) is one such package that tries to evaluate the sentiments and emotions associated with texts [22]. The sentimentr package has been successfully used in analyzing the sentiments of tweets on migraine activity [23]. It has been also used to analyze the tweets of Donald Trump to examine the relation between tweet sentiment and the number of retweets [24]. In a review of four different sentiment computation packages, Naldi [25] concluded that the critical issue of negators is accurately dealt in the sentimentr package. In other words, sentimentr was accurate in calculating the difference between words like “useful,” “not useful” (negator), “really useful” (amplifier), and “hardly useful” (deamplifier). The potential of this package to calculate the sentiments based on the role of negators, amplifiers, and deamplifiers was the reason this package was used to analyze tweet sentiments in this study.

**Figure 1:**
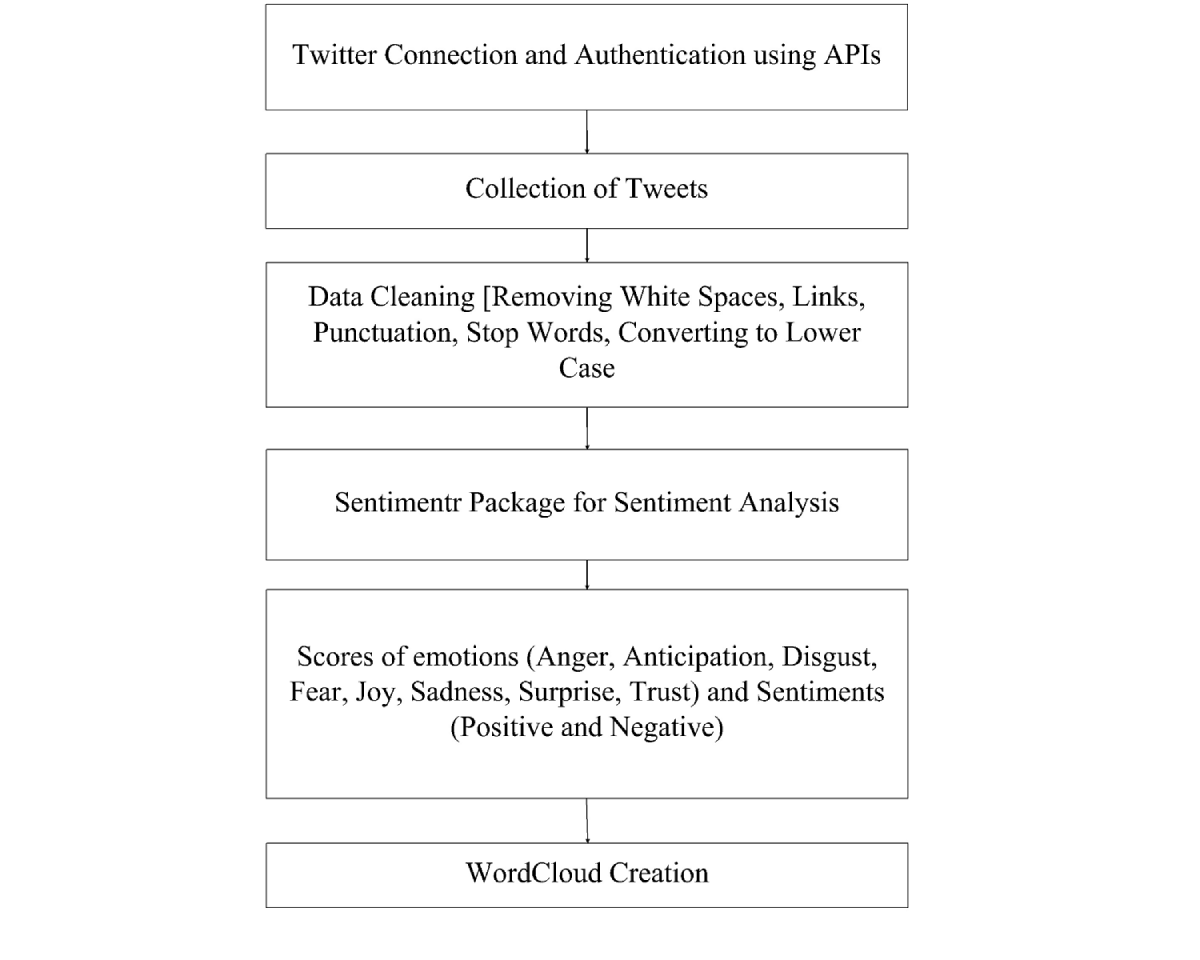
Flowchart for sentiment analysis of tweets. API: application programming interface.

Figure 1 illustrates the flowchart for this study’s sentiment and emotion analysis of tweets. The tweets were collected by using rtweet package in R (The R Foundation). To collect tweets, the *search_tweets* function of rtweet was used. The following keywords were used to fetch tweets during the collection process: #ChineseVirus, #ChineseVirusCorona, and #WuhanVirus. The date range of the search was set to April 11-16, 2020. The search process did not collect retweets and replies, so that the duplication of data can be avoided.

After the tweets were collected, the data cleaning process was performed using the Text Mining package in R. This package was used to remove white space, punctuation, stop words, and the tweets were converted to lower case. After data cleaning, the sentimentr package was applied to analyze the tweets. Once the scoring of the tweets was done on the basis of sentiments and emotions, the terms related to positive and negative sentiments, profanity, and emotions were also calculated for further analysis.

## Results

Using the tweet collection process, a total of 16,000 tweets were collected for the analysis. The collected tweets were analyzed using the sentimentr package in R, and the scoring was done on the basis of positive and negative sentiments. The sentimentr package scores sentiments on a scale where 0 is considered neutral, negative numbers indicate the presence of negative sentiments, and positive numbers indicate the presence of positive sentiments. The sentiment score of each tweet was calculated individually and then the complete report of the sentiment across all tweets was generated.

The minimum value obtained in the analysis is –1.930, which is the score of the tweet with the most negative sentiment. The maximum score obtained during the analysis is 5.371 (ie, the most positive tweet). The median and mean of the sentiments are –0.016 and –0.063, respectively. This shows that the sentiments observed in the tweets have a negative skew, that is, the number of tweets with negative sentiments were more prevalent than the number of positive sentiments.

Table 1 shows the emotion analysis of the collected tweets. While the sentiment analysis of the tweets provide an overview of how people were tweeting, the emotion analysis provides insight into why this was happening. It can be seen that tweets expressing fear are almost equal in prevalence to the tweets related to trust. When the four negative emotions (fear, sadness, anger, and disgust) were analyzed collectively, they comprised 52.18% (n=8450) of the sample. While this result confirms the presence of primarily negative sentiments in the tweets sampled, it also discloses the constituents of the negative sentiments in the tweets. Sample tweets expressing different emotions are shown in Table 2.

**Table 1:** Emotion analysis of tweets.

Emotion	Tweets, n (%)
Trust	2926 (18.29)
Fear	2857 (17.86)
Sadness	2123 (13.27)
Anticipation	2005 (12.53)
Anger	1972 (12.32)
Disgust	1498 (9.36)
Joy	1422 (8.89)
Surprise	1198 (7.49)

**Table 2:** Sample tweets with different emotions.

Emotion	Tweet
Trust	“CMgovt has done very good job to handle #WuhanVirus problem in state” “Breaking News US Government gave Wuhan Virology lab aMillion grant for virus research #CoronaVirusUpdate #Coronavirus #COVID #Covid #WuhanVirus #BreakingNews” “Great now Biden needs to explain that him calling the Chinese travel ban xenophobic would NEVER happen again and he made a HUGE mistake cause this attitude would kill us all if he was president #ChineseVirusCorona”
Anger	“Good for Trump Calling the virus what it is the Wuhan virus Dont know why it triggers you so badly but you always have to find something daily to bang Trump around It gets old and you look childish #WuhanVirus #WuhanFlu” “Chinas carelessness and deceit has crashed global economies and costed countries trillions of dollars Every cent of debt that they hold from other countries should be forgiven Coronavirus #WuhanVirus #ChinaLiedPeopleDied” “I guess Twitter should ban all the Chinese because its ban in China #ChinaLiedPeopleDied #ChineseVirus”
Sadness	“Sir we had great hopes with you but it all shattered into pieces You r siding with the evil of CCP in this difficult timeshas become a mouthpiece for communist chinafunding it would be a crime against humanity #ChineseVirus” “Just look at the economic destruction the #WuhanVirus has inflicted on the American ppl” “#ChineseVirus COVID We lost th member of familyfriend in the CoronaWarfound ve hospitalised Yesterday world heard news of a mths old corona infected baby I plead to my friendsfollowers Corona is a seriousreal #KeepSocialDistancing #StayHomeSaveLives”
Anticipation	“Considering most of the vaccines across globe come from India India might play a vital role in discovery of vaccine on #ChineseVirus #VaccinesSaveLives” “Our life is going to be changed drasticallyBe prepared for it#ChineseVirus” “I KNEW IT I think all countries should declare war on China WHO #ChinaLiedPeopleDie #Wuhan #ChineseVirus #ChinaMustPay #ChinaVsTheWorld”
Fear	“We are heading for a long haul I am afraid in this fight against #ChineseVirus with this pandering to a community which was not supposed to be in India since” “We are afraid of #ChineseVirus so we are retreating now” “Sorry i afraid of #ChineseVirusCorona #ChineseVirus”
Disgust	“Will you be so shameless to buy Chinese mobile phones after #ChinaVirus #ChineseVirusCorona #coronavirusindia #coronavirus #COVID #covidindia Hit China hard where it will pain them most PS A branded phone fetches Chinese company more profit than selling components” “Shame on you #ChineseVirusCorona” “Shameful the very same people who caused the #WuhanVirus pandemic is now discriminating against innocent Africans”
Joy	“Happy Thai New Year Buddy Lets fight together #MilkTeaAlliance #FightForFreedom #StandwithHK #hkisnotchina #TaiwanIsaCountry nnevvyy #ChineseVirus #ChinaMustPay #ChinaLiedAndPeopleDied” “Not only recovered but got raised big fundLovely D coronavirus #ChineseVirus #WuhanVirus” “I get enough to live comfortably This #ChineseVirus just depleted my savings Im happy with my investment”
Surprise	“Am surprised we still trust China havent we learnt our lesson #ChineseVirus” “Shocking Did you know your taxwere being spent on this So is NIH partially responsible for #WuhanVirus” “The world is still in utter shock. Right from the start experts advised the president to refrain from labelling COVIDa #ChineseVirus to no avail It appears whatever DonaldJTrump was harboring against China has finally started manifesting in life threatening developments”

While analyzing the tweets, the 15 most frequent words conveying different emotions were also analyzed. The results of the analysis are illustrated in Table 3. Words like death, good, money, pay, pandemic, Trump, and organization were most frequently used by people while mentioning terms like “ChineseVirus,” “WuhanVirus,” and “ChineseVirusCorona.” The presence of words like death, pay, pandemic, evil, and disease were repeatedly used in the tweets associated with negative sentiments and emotions. These results, combined with Table 3 and the statistics presented earlier, reflect the negative sentiments and emotions that have been communicated online.

**Table 3:** Frequency of the most used terms in the analyzed tweets.

Term	Tweets, n (%)
Death	1656 (13.51)
Good	1352 (11.03)
Money	1305 (10.65)
Pay	1284 (10.48)
Pandemic	1254 (10.23)
Trump	824 (6.72)
Organization	668 (5.45)
Hope	588 (4.80)
God	536 (4.37)
Time	513 (4.19)
Evil	472 (3.85)
Bad	472 (3.85)
Fight	470 (3.83)
Medical	447 (3.65)
Disease	416 (3.39)

## Discussion

### Principal Findings

Based on the results obtained in the analysis, the negative sentiments and emotions associated with the collected tweets are evident. A good number of tweets including the term “Chinese Virus” expressed hatred, disgust, fear, and anger. Apart from the words that were associated with different emotions, there were some slang words or constructions created by users that were not detected by the sentimentr package. Prominent among those were “ccpisterrorist,” “ccpliedpeopledied,” “ccpvirus,” “ccpviruscoronavirus,” “chinaliedpeopledied,” “chinamustexplain,” “chinamustpay,” “chinesebioterrorism,” “kungflu,” “makechinapay,” “milkteaalliance,” “wholiedpeopledied,” “wuhanhealthorganisation.” The “ccp” in these terms refers to the Chinese Communist Party, the ruling party of China headed by Xi Jinping, the President of the People’s Republic of China. Some of these terms also expressed anger toward the WHO, calling it “Wuhan Health Organization.” This trend suggests that both China and the WHO are being held responsible for the spread of COVID-19.

Prominent words like virus, Trump, pandemic, government, outbreak, pay, communist, propaganda, blame, killed, shame, killing, shit, hell, stupid, lying, lies, die, etc, were used to reflect negative sentiments in tweets, while words like right, like, good, money, accountable, humanity, responsible, work, organization, great, better, well, global, please, thanks, etc, were used to indicate positive sentiments in tweets. During the analysis, terms categorized as profanity were also analyzed, and a frequent usage of profane words in tweets was observed. This list includes fuck, shit, hell, fucking, ass, crap, screw, fucks, bastards, bitch, bastard, Nazis, ahole, and nazi. These words reflect the disgust-related emotions in tweets.

Overall, based on the findings of this paper, it can be clearly stated that the sentiments of people tweeting about the so-called “Chinese Virus” have been mostly negative. The use of negative words, combined with a good dosage of profane terms, reflect the emotions of tweets, which are mainly concentrated toward a sense of fear, sadness, anger, and disgust. The results also indicate signs of discrimination and racism in the COVID-19 era, which has been previously shown by Coates [26]. The results obtained in this study further strengthen the fact that there has been a substantial increase in cyber racism due to COVID-19.

### Conclusion and Future Works

In this paper, tweets were analyzed to evaluate the level of cyber racism encountered during the COVID-19 pandemic. For this purpose, tweets were collected if they mentioned “ChineseVirus,” “WuhanVirus,” or “ChineseVirusCorona.” This work demonstrates that the sentiments of a majority of the tweets were negative. Further analysis of emotions associated with the tweets also revealed that there was a sense of fear, anger, and disgust among Twitter users. Additionally, there were also slang terms that expressed negative sentiments toward China, Wuhan, and the WHO. The majority of the terms used in the tweets were negative and included death, pay, communist, ccp, racist, etc. The study also revealed a substantial use of profane words, which supports the conclusion that cyber racism has been increasing during the COVID-19 pandemic. Future studies can build on this study by analyzing trends of cyber racism in the coming days.

## Abbreviations

PHEIC: Public Health Emergency of International Concern
WHO: World Health Organization
